# Clinical characterization and founder effect analysis in Chinese amyotrophic lateral sclerosis patients with *SOD1* common variants

**DOI:** 10.1080/07853890.2024.2407522

**Published:** 2024-10-01

**Authors:** Pei-Shan Wang, Xin-Xia Yang, Qiao Wei, Yong-Ting Lv, Zhi-Ying Wu, Hong-Fu Li

**Affiliations:** a Department of Medical Genetics and Center for Rare Diseases, Second Affiliated Hospital, Zhejiang University School of Medicine, Hangzhou, China; b Department of Neurology and Key Laboratory of Medical Neurobiology of Zhejiang Province, Zhejiang University School of Medicine, Hangzhou, China; c Nanhu Brain-computer Interface Institute, Hangzhou, China; d MOE Frontier Science Center for Brain Research and Brain-Machine Integration, Zhejiang University, Hangzhou, China

**Keywords:** Amyotrophic lateral sclerosis, *SOD1*, Chinese, common variants, founder effect, SOD1 aggregation

## Abstract

**Objective:**

In the Asian population, *SOD1* variants are the most common cause of amyotrophic lateral sclerosis (ALS). To date, more than 200 variants have been reported in *SOD1*. This study aimed to summarize the genotype–phenotype correlation and determine whether the patients carrying common variants derive from a common ancestor.

**Methods:**

A total of 103 sporadic ALS (SALS) and 11 familial ALS (FALS) probands were included and variants were screened by whole exome sequencing. Functional analyses were performed on fibroblasts derived from patients with *SOD1* p.V48A and control. Haplotype analysis was performed in the probands with p.H47R or p.V48A and their familial members.

**Results:**

A total of 25 *SOD1* variants were identified in 44 probands, in which p.H47R, p.V48A and p.C112Y variants were the most common variants. 94.3% and 60% of patients with p.H47R or p.V48A had lower limb onset with predominant lower motor neurons (LMNs) involvement. Patients with p.H47R had a slow progression and prolonged survival time, while patients with p.V48A exhibited a duration of 2–5 years. Patients with p.C112Y variant showed remarkable phenotypic variation in age at onset and disease course. *SOD1^V48A^* fibroblasts showed mutant SOD1 aggregate formation, enhanced intracellular reactive oxygen species level, and decreased mitochondrial membrane potential compared to the control fibroblast. Haplotype analysis showed that seven families had two different haplotypes. p.H47R and p.V48A variants did not originate from a common founder.

**Conclusions:**

Our study expanded the understanding of the genotype–phenotype correlation of ALS with *SOD1* variants and revealed that the common p.H47R or p.V48A variant did not have a founder effect.

## Key Messages

- In our ALS cohort, 44 ALS probands were identified with 25 *SOD1* variants, of which p.H47R, p.V48A and p.C112Y variants were the most frequent. The genotype–phenotype relationship of patients with *SOD1* p.H47R, p.V48A and p.C112Y patients were summarized.
- *SOD1^V48A^* fibroblasts showed mutant SOD1 aggregate formation, enhanced intracellular reactive oxygen species level, and decreased mitochondrial membrane potential compared to the control fibroblast.
- Our study expanded the understanding of the genotype–phenotype correlation of ALS with *SOD1* variants and showed the common variants p.H47R or p.V48A did not have a founder effect.

## Introduction

Amyotrophic lateral sclerosis (ALS) is a neurodegenerative disease characterized by progressive muscle weakness and atrophy due to the deterioration of upper motor neurons (UMNs) and lower motor neurons (LMNs) [1]. Tragically, most patients were deceased within 3–5 years due to respiratory failure. The exact mechanisms underlying ALS remain elusive by now. The disease is typically categorized into familial and sporadic forms. Familial ALS (FALS) constitutes 5–10% of cases where multiple occurrences are observed within a family, while the sporadic ALS (SALS) was more prevalent [2]. Since the identification of *SOD1* variants in FALS patients in 1993 [3], the genetic landscape of ALS has expanded to encompass more than 40 associated genes [4]. It is estimated that approximately 70% of FALS and 15% of SALS harbour such genetic variants [2], with *SOD1* variants being particularly prevalent among Asian population, notably in FALS [5]. To date, more than 200 variants have been reported in *SOD1* (http://www.hgmd.cf.ac.uk/). Intriguingly, these diverse *SOD1* variants give rise to a broad spectrum of clinical manifestations in ALS patients. For instance, the p.D11Y variant is characterized by distal limb onset and a slow course [6]. Conversely, patients with homozygous p.D91A show an insidious onset and a slow progression with bladder involvement at the later stage, diverging from heterozygous p.D91A carriers, whose symptoms can greatly vary [7]. Individuals harbouring p.A5V variant exhibit an aggressive limb-onset phenotype and survive less than two years [8]. Patients with p.G42S present as a severe spinal onset, progressing to bulbar involvement and culminating in a brief survival period, typically around one year [9]. In contrast, patients with p.E101G or p.G94C demonstrate prolonged survival [7,10]. Thus, elucidating the intricate link between *SOD1* genotypes and their respective phenotype is pivotal for advancing our comprehension of ALS complex nature.

The *SOD1* gene encodes superoxide dismutase-1, one of three superoxide dismutase enzymes found in humans. *SOD1* is ubiquitously expressed and provides a defence against oxygen toxicity by leveraging its copper-zinc bound, highly stable homodimer structure. However, mutated SOD1 leads to deleterious alterations, manifesting as conformational abnormalities, aggregation, mitochondrial dysfunction and prion-like propagation [11]. The prevailing hypothesis in SOD1-associated ALS implicates detrimental gain-of-function mechanisms as central to pathogenesis [12].

Geographical disparities exist in the prevalence of *SOD1* variants. For example, the p.A5V is the most common in North America [13], while p.I114T dominates in the United Kingdom and p.L145F in Italy [14, 15]. In our previous study, we found that *SOD1* p.H47R and p.V48A variants were most frequent in Southeastern China [16]. Given the close genetic proximity of the p.H47R and p.V48A variant sites, their potential shared ancestral origin remains unexplored. Hence, the aim of this study is to determine whether the subjects carrying the p.H47R or p.V48A variants share a common founding lineage. Complementary to this, functional analyses were performed to ascertain the pathogenicity of the p.V48A variant. Additionally, we summarized the genotype–phenotype correlation of these three variants, deepening our insights into the pathology of ALS.

## Materials and methods

### Subjects

A total of 103 SALS and 11 FALS were recruited from the Second Affiliated Hospital of Zhejiang University School of Medicine, from May 2021 to March 2023. For further genotype–phenotype analysis, we included previously reported *SOD1*-mutated probands in our centres from December 2007 to April 2021. Each patient was diagnosed with ALS by two senior neurologists fulfilling the Gold Coast diagnostic criteria [17]. Electromyography (EMG) was performed on all patients. Blood samples from available family members were also obtained. This study was approved by the Ethics Committee of the Second Affiliated Hospital, Zhejiang University School of Medicine (approval no. 2015-IRB-045). Written informed consent was obtained from all participants.

### Genetic analyses and Sanger sequencing

We extracted genomic DNA from participants’ peripheral blood samples by using QIAamp blood genomic extraction kits (Qiagen, Hilden, Germany) following the standard protocols. Whole exome sequencing (WES) was performed by Agilent SureSelect Human All Exome V6 kit (Agilent Technologies Inc., Santa Clara, CA). The captured reads were sequenced on the Illumina HiSeq X Ten platform (XY Biotechnology Co. Ltd., Hangzhou, China), and variants were annotated with ANNOVAR software. The frequency of the identified variants in the general population was estimated by Genome Aggregation Database (gnomAD), Exome Aggregation Consortium (ExAC) database and the 1000 Genomes Project (1000G) database. The change of protein function was predicted by Sorting Intolerant from Tolerant (SIFT, https://sift.bii.a-star.edu.sg/), PolyPhen-2 (http://genetics.bwh.harvard.edu/pph2/), Combined Annotation Dependent Depletion (CADD, https://cadd.gs.washington.edu/) and Mutation Taster (http://www.mutationtaster.org/). Sanger sequencing was carried out to validate the variant and co-segregation in each proband and available familial members.

### Primary fibroblasts culture

Primary fibroblast cell lines were established from a 4-mm skin biopsy from the proband of family 7, 8 and 10, and from a healthy control. Fibroblast cells were grown in DMEM (Gibco, Waltham, MA) supplemented with 10% foetal bovine serum (Gibco, Waltham, MA), and 1% penicillin/ampicillin antibiotics (Gibco, Waltham, MA) at 37 °C, 5% CO_2_.

### Immunofluorescence microscopy

The fibroblasts were cultured in 24-well glass slides (NEST) and fixed with 4% paraformaldehyde for 15 min at room temperature. After being washed three times with ice-cold phosphate-buffered saline (PBS), fibroblasts were permeabilized with 0.3% Triton X-100, and blocked with 5% bovine serum albumin (BSA; Sigma, St. Louis, MO) in PBS for 1 h. Fibroblasts were incubated with anti-SOD1 (1:100; Abcam, Cambridge, UK) in blocking solution at 4 °C overnight, followed by secondary antibody anti-rabbit IgG Alexa Fluor 488 (1:1000; Life Technologies, Carlsbad, CA). Cell nuclei were stained with 4′,6-diamidino-2-phenylindole (DAPI, 1:1000; Roche, Basel, Switzerland). Fluorescence images were captured by Zeiss LSM 900 confocal system (Oberkochen, Germany).

### Reactive oxygen species (ROS) and mitochondrial membrane potential (MMP) assay

The ROS assay was performed using a Reactive Oxygen Species Assay Kit (Beyotime Biotechnology, Beijing, China). Fibroblasts were digested by trypsin and incubated with DCFH-DA (1:1000) diluting in DMEM for 20 min at 37 °C in the dark. Next, wash cells three times with DMEM, centrifuging each time at 600 × *g* for 4 min at 4 °C. The intracellular ROS level was detected by flow cytometry (FCM).

The MMP assay was performed using an enhanced mitochondrial membrane potential assay kit with JC-1 (Beyotime Biotechnology, Beijing, China) according to the manufacturer’s instructions. Fibroblasts were digested by trypsin and incubated with JC-1 (1:200) diluting in 1 × JC-1 staining buffer for 20 min at 37 °C in the dark, followed by washing three times with 1 × JC-1 staining buffer. Changes in intracellular MMP were detected by FCM.

### Haplotype analysis

A set of 13 single nucleotide polymorphisms (SNPs) in chromosome 21 inside and around *SOD1* was selected from previous studies to investigate the origin of the p.H47R and p.V48A variants [8]. Haplotype analysis was performed using PCR amplification and Sanger sequencing. Their primers are shown in Supplementary Table S1.

### Statistical analyses

Data are presented in the figures as the mean ± standard deviation. Statistical analyses were performed using Student’s *t*-test in GraphPad Prism 9 software (La Jolla, CA). Statistical significance was defined as *p* value <.05. Differences were considered statistically significant at *p < .05, **p < .01 or ***p < .001.

## Results

### Genetic findings and variant spectrum in our ALS patients

From 2021 to 2023, 21 variants were detected in ALS patients. In 11 FALS cases, *SOD1* variants accounted for 36.4% (4/11) followed by *FUS* variants (2/11, 18.2%). In 103 SALS cases, *SOD1* variants accounted for 7.8% (8/103) followed by *TARDBP* variants (4/103, 3.9%). Only one patient of each carried the *TBK1*, *DCTN1* or *UBQLN2* variant.

**Figure 1.**
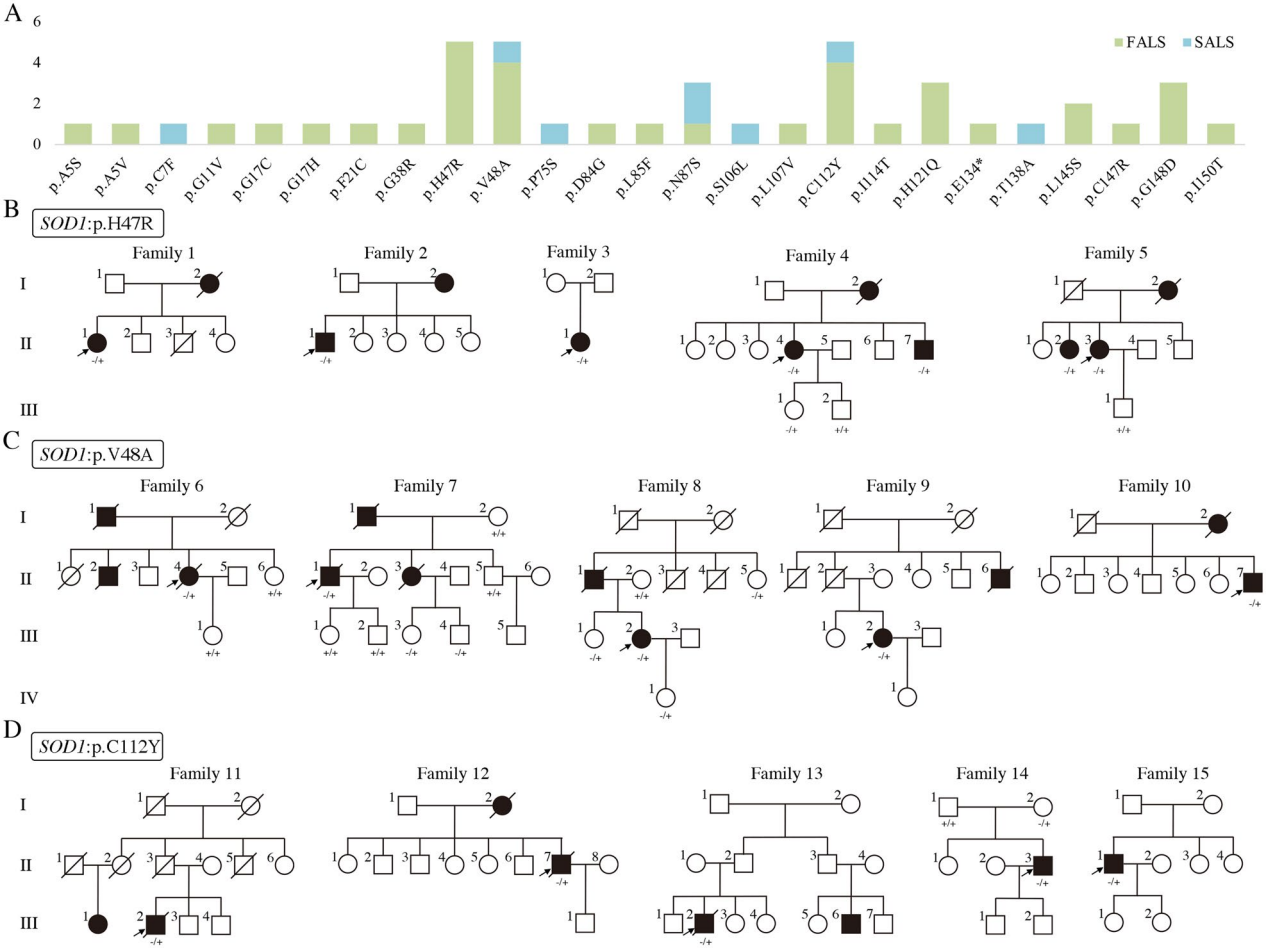
*SOD1* variant distribution and pedigrees of patients with *SOD1* p.H47R, p.V48A, p.C112Y variant. (A) Variant spectrum of *SOD1* in Southeastern Chinese ALS patients. (B) Pedigrees of patients with *SOD1* p.H47R. (C) Pedigrees of patients with *SOD1* p.V48A. (D) Pedigrees of patients with *SOD1* p.C112Y. Squares indicate male, circles indicate female, solid symbols indicate affected individuals and arrows indicate the probands.

A total of 12 patients were identified to carry *SOD1* variants, including p.C7F, p.H47R, p.V48A, p.L85F, p.N87S, p.S106L, p.C112Y, p.H121Q and p.T138A. Four of them were FALS (4/12, 33.3%) and the else were SALS (8/12, 66.7%). Combining our previous results, a total of 25 *SOD1* variants were identified in 44 probands (Supplementary Table S2). Most variants were distributed in exons 1, 4 and 5 (22/25, 88.0%), and most patients presented with spinal onset (42/43, 97.7%). FALS accounts for 77.3% (34/44) of *SOD1*-ALS patients and SALS accounts for 22.7% (10/44). Variants in p.H47R, p.V48A and p.C112Y were found in each of the five probands (5/44, 11.4%), which were the most common variants (Figure 1(A)).

### Clinical features of probands carrying *SOD1* p.H47R, p.V48A or p.C112Y

#### Probands with SOD1 p.H47R variant

Five probands in our cohort were identified to carry *SOD1* p.H47R variant (Figure 1(B)), of which four were female. All probands presented symptoms in their fifties. Proband 1 (family 1, II-1) and proband 4 (family 4, II-4) were upper limb onset. They had a slow progression and were diagnosed with ALS according to neurological examinations and EMG at least 3 years after onset. Proband 2 (family 2, II-1), proband 3 (family 3, II-1) and proband 5 (family 5, II-3) developed initial symptoms of lower limb weakness and were diagnosed with ALS with a two-year diagnosis delay. Four probands except proband 4 exhibited predominantly LMN features. Three probands had intact bulbar and respiratory functions during the follow-up period. All probands had no sensory and cognition impairment.

#### Probands with SOD1 p.V48A variant

Five other probands in our cohort were detected to carry *SOD1* p.V48A variant (Figure 1(C)). Three of them were female and all of them had a positive family history. Proband 6 (family 6, II-4), proband 7 (family 7, II-1), proband 9 (family 9, III-2) and proband 10 (family 10, II-3) initially presented with weakness and atrophy of the lower limb and had bulbar function impairment in the subsequent disease course, exhibiting dysarthria and dysphagia. Proband 8 (family 8, III-2) initially exhibited upper limbs weakness, which gradually spread throughout her whole body. In these probands, neurological examinations showed decreased muscle strength in extremities and EMG revealed acute and chronic neurogenic denervation in the affected regions. Probands 6–9 showed predominant LMN involvement. All probands were diagnosed within 2 years after the onset. Probands 6 and 7 died of respiratory failure.

#### Probands with SOD1 p.C112Y variant

Five probands were detected to carry *SOD1* p.C112Y variant (Figure 1(D)). All of them were male and 80% of them had a positive family history. Proband 12 (family 12, II-7) initially exhibited upper limb weakness and the other four probands had lower limb onset. All probands clinically presented with predominant LMN signs and were diagnosed within 2 years after the onset. The bulbar symptom was found in probands 11, 12, 14 and 15. Probands 11 and 12 were deceased due to respiratory failure.

### Genetic findings and genotype–phenotype correlation

Apart from 15 ALS probands in our cohort, the p.H47R variant was also identified in two siblings (family 4: II-7, family 5: II-2) and an asymptomatic subject (family 6: III-1), while the p.V48A variant was also identified in five asymptomatic subjects (family 7: III-3, III-4, family 8: II-5, III-1, IV-1) and the p.C112Y variant was found in an asymptomatic subject (family 14: I-2). The three asymptomatic carriers observed in family 8 and family 14 suggested an incomplete penetrance of p.V48A and p.C112Y variants.

After reviewing previous literature [10,18–40], we found that major pedigrees with *SOD1* p.H47R were from Asia (25/34, 73.5%) and 82.1% of pedigrees had a positive family history (Supplementary Table S3). The vast majority of patients (83/88, 94.3%) had a lower limb onset with predominant LMN involvement. Correspondingly, patients with p.H47R had a slow progression and prolonged survival time. Besides, all of the patients carrying *SOD1* p.V48A were Chinese, and 50% of the patients had a positive family history (Table 1). Age at onset ranged from 42 to 64 years old and the majority of patients (6/9, 66.7%) were presented with spinal onset. LMN involvement was the main manifestation (4/5, 80%). The life expectancies varied greatly and the mean disease duration was 41.4 ± 8.2 months. 88.9% of pedigrees with *SOD1* p.C112Y were from China and more than half of pedigrees (11/17) were FALS (Table 2). Age at onset ranged from 20s to 70s and the disease duration varied from one year to >69 years. Bulbar symptoms were frequently observed during the progression of the disease (9/15, 60%).

**Table 1.** Clinical features of patients carried *SOD1* p.V48A.

Pedigree	Familial distribution	Country	Family history	Gender	AAO, year, mean ± SD	Onset site	Diagnostic delay, months, median (range)	Phenotype	Bulbar symptom	Respiratory failure	Cognitive impairment	Disease duration, month, mean ± SD	Reference
P1	Proband	China	Yes	F	53	LL	20	LMN dominance	+	+	−	31	Present study
P2	Proband	China	Yes	M	64	LL	12	LMN dominance	+	+	−	26	Present study
P3	Proband	China	Yes	F	42	UL	12	LMN dominance	−	−	−	>53	Present study
P4	Proband	China	Yes	F	46	LL	13	LMN dominance	+	+	−	>48	Present study
P5	Proband	China	Yes	M	49	LL	15	Classical ALS	+	−	−	>18	Present study
P6–7	Proband	China	No	1M/1F	51.5 ± 3.5	Bulbar/limbs	15.5 (6–25)	NA	NA	NA	NA	60 and NA	Tang et al. [21]
P8	Proband	China	No	M	54	Bulbar	NA	NA	NA	NA	NA	NA	Edgar et al. [31]
P9	Proband	China	No	M	56	Bulbar	NA	NA	NA	NA	NA	NA	Edgar et al. [31]
P10	Proband	China	No	M	63.5	NA	NA	NA	NA	NA	NA	5.1	Chen et al. [20]
P11	Proband	China	NA	NA	NA	NA	NA	NA	NA	NA	NA	NA	Chen et al. [32]

AAO: age at onset; M: male; F: female; SD: standard deviation; LL: lower limb; UL: upper limb; LMN: lower motor neuron; ALS: amyotrophic lateral sclerosis; NA: not available.

**Table 2.** Clinical features of patients carried *SOD1* p.C112Y.

Pedigree	Familial distribution	Country	Family history	Gender	AAO, year, mean ± SD	Onset site	Diagnostic delay, months, median (range)	Phenotype	Bulbar symptom	Respiratory failure	Cognitive impairment	Disease duration, month, mean ± SD	Reference
P1	Proband	China	Y	M	46	LL	6	LMN dominance	+	+	−	60	Present study
P2	Proband	China	Y	M	47	UL	9	LMN dominance	+	+	−	53	Present study
P3	Proband	China	Y	M	50	LL	15	LMN dominance	NA	NA	−	40	Present study
P4	Proband	China	Y	M	29	LL	8	LMN dominance	+	−	−	>20	Present study
P5	Proband	China	N	M	52	LL	10	LMN dominance	+	−	−	>16	Present study
P6	Proband	China	NA	F	70	UL	NA	NA	NA	NA	NA	30	Liu et al. [33]
P7	Proband	China	Y	M	59	LL	NA	Classical ALS	NA	NA	NA	NA	Eisen et al. [34]
P8	Proband	China	Y	F	68	UL	NA	Classical ALS	−	NA	NA	NA	Hou et al. [35]
P9	Proband	China	N	M	NA	UL and LL	NA	Classical ALS	+	NA	NA	NA	Hou et al. [35]
P10	Proband	China	Y	F	70	UL	20	NA	NA	+	NA	48	Liu et al. [36]
P11–12	Proband	China	1Y/1N	1M/1F	27.5 ± 10.6	Bulbar(1)/limbs(1)	6/74	NA	NA	NA	NA	72.0 ± 72.1(2)	Tang et al. [21]
P13	Proband	China	Y	M	38.8	LL	11	NA	NA	NA	NA	>39	Wei et al. [37]
P14	Proband	China	N	M	26.7	NA	NA	NA	NA	NA	NA	16.6	Chen et al. [20]
P15	Proband	China	N	M	38.8	NA	NA	NA	NA	NA	NA	40.1	Chen et al. [20]
P16	4 patients in 1 generation	China	Y	3M/1F	53.8 ± 8.4(4)	UL(1)/LL(3)	NA	LMN dominance	NA	NA	NA	19.2 ± 7.2(9)	Li et al. [38]
P17	Proband	Poland	N	M	43	UL	8	Classical ALS	NA	NA	NA	NA	Berdyński et al. [39]
P18	9 patients in 3 generations	Japan	Y	6M/3F	39.0 ± 13.7(9)	Bulbar(2)/UL (4)/LL(3)	NA	LMN dominance	4	NA	NA	172.2 ± 307.1(9)	Nakamura et al. [40]

AAO: age at onset; M: male; F: female; SD: standard deviation; Y: yes; N: no; LL: lower limb; UL: upper limb; LMN: lower motor neuron; ALS: amyotrophic lateral sclerosis; NA: not available.

### Mutant SOD1 protein aggregates in *SOD1^V48A^* fibroblast cells

**Figure 2.**
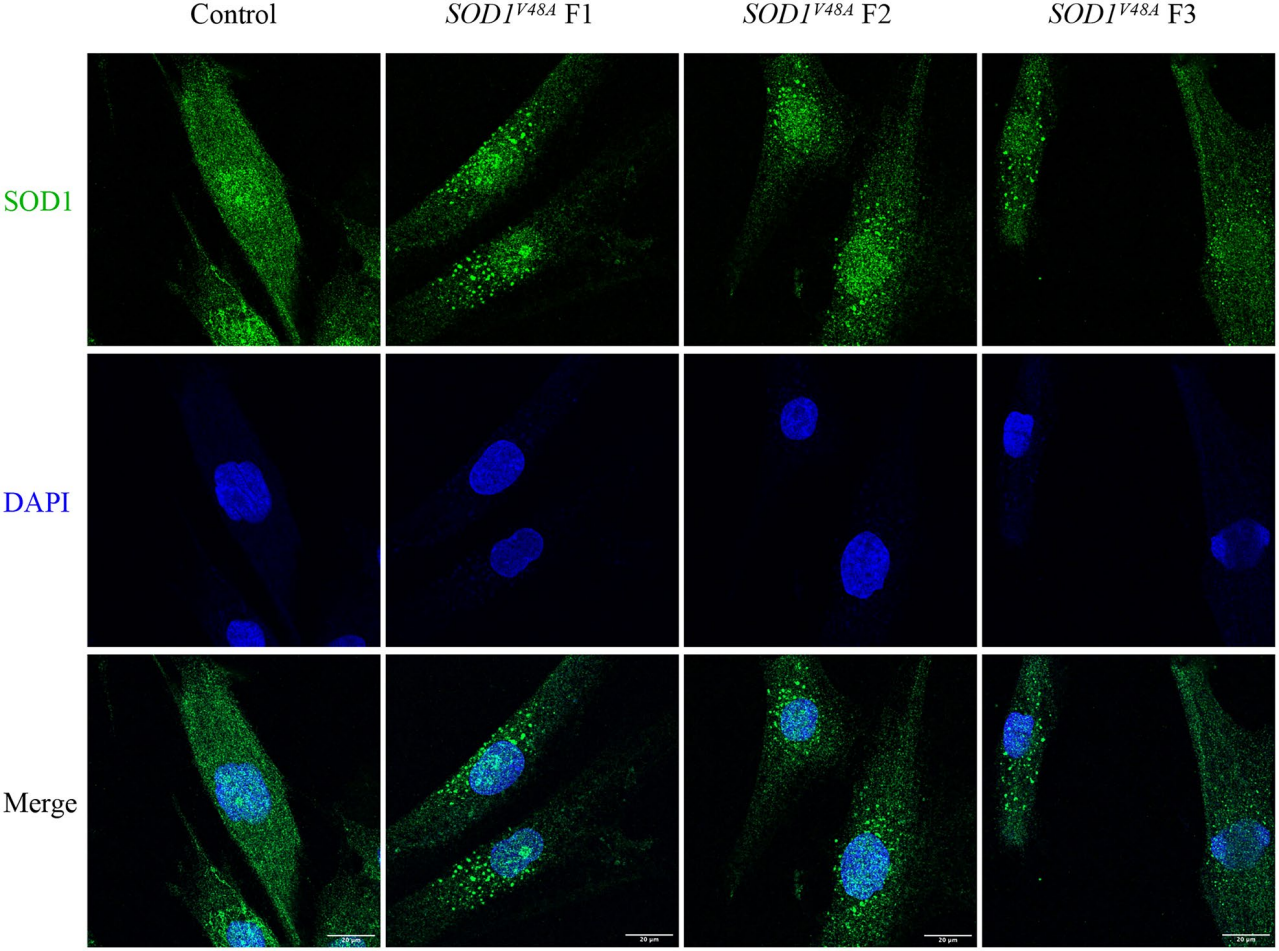
Mutant SOD1 aggregates formation by immunofluorescence. Immunofluorescence in *SOD1^V48A^* fibroblasts and control fibroblast labelled with antibodies against SOD1 (green). *SOD1^V48A^* F1, F2 and F3 were derived from the proband of family 7, 8 and 10, respectively. Cell nuclei were stained with 4′,6-diamidino-2-phenylindole (DAPI, blue). Scale bars: 20 μm.

Variants in *SOD1* lead to abnormal protein folding and aggregate formation. To determine whether p.V48A variant leads to aggregates, we performed immunofluorescence using the patient’s fibroblasts. The immunofluorescence result showed that the wide-type SOD1 protein was widely distributed within cells. However, in the *SOD1^V48A^* fibroblasts (F1, F2 and F3), SOD1 aggregates were observed in the cytoplasm and some of them were perinuclear while absent in the control fibroblast (Figure 2).

### Mitochondrial dysfunction in *SOD1^V48A^* fibroblast cells

**Figure 3.**
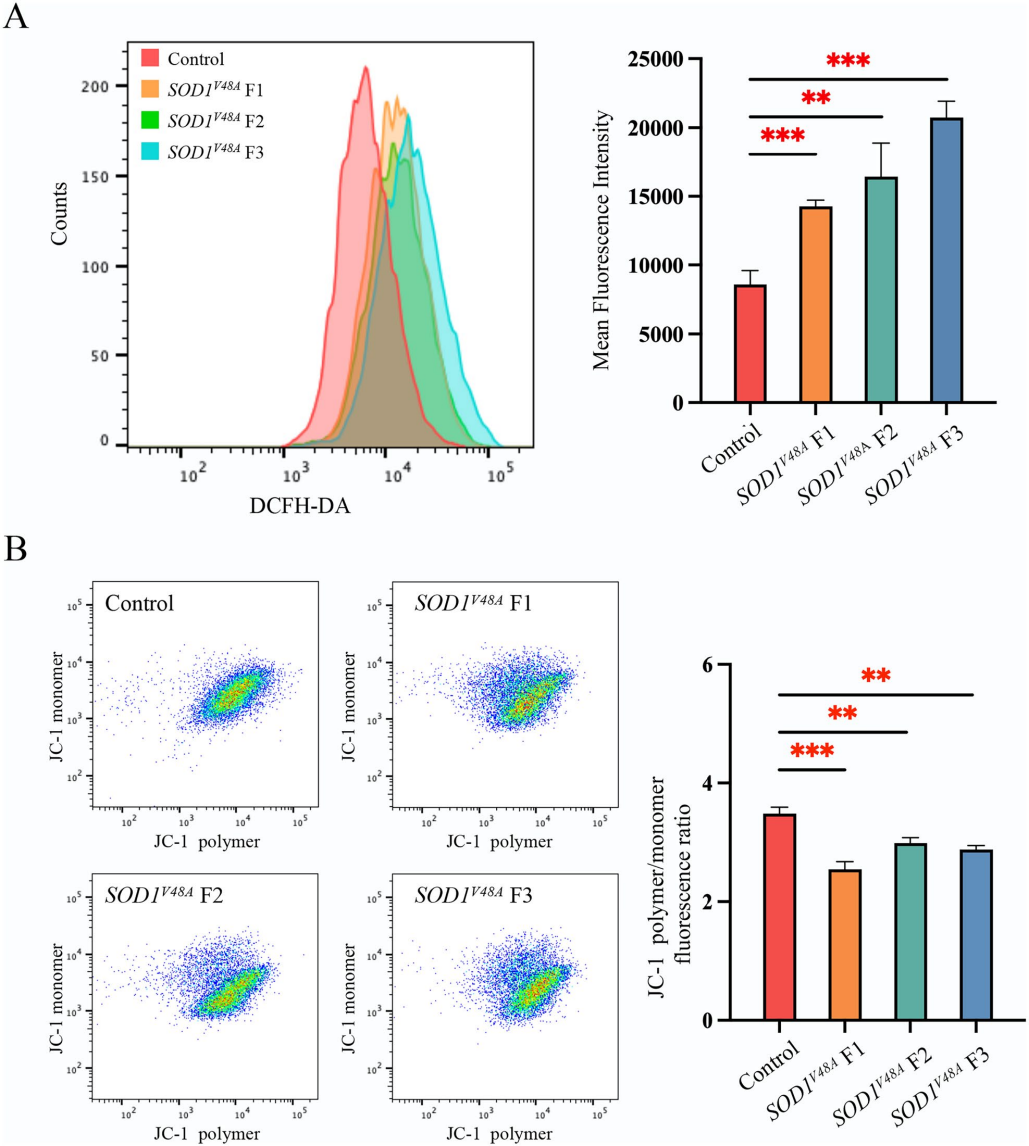
*SOD1^V48A^* fibroblasts exhibit mitochondrial dysfunction. (A, B) Detection of the total intracellular ROS levels and MMP change. *SOD1^V48A^* fibroblasts F1, F2 and F3 were derived from the proband of family 7, 8 and 10, respectively. ROS level was measured by mean fluorescence intensity and MMP was measured by the relative fluorescence density of the red/green ratio. Each experiment was repeated three times. Error bars indicate means ± SD. **p < .01 or ***p < .001 by Student’s *t*-test.

As mitochondrial dysfunction and oxidative stress are associated with ALS pathogenesis, to further analyse the pathogenicity of this variant, we evaluated intracellular ROS level and MMP. We used a green fluorescent DCFH-DA probe to detect intracellular ROS generation. As shown in Figure 3(A), green fluorescence was enhanced in all *SOD1^V48A^* fibroblasts (F1, F2 and F3) compared to the control. The measurements of JC-1 fluorescence showed enhanced green fluorescence intensity and decreased red fluorescence intensity in *SOD1^V48A^* fibroblasts (F1, F2 and F3) compared with the control (Figure 3(B)).

### Haplotype analysis

In order to evaluate whether the patients carrying the p.H47R or p.V48Avariants derive from a common ancestor, we genotyped 13 SNPs around the *SOD1* gene in probands and their family members. The results showed there are two shared haplotypes and the size of the shared haplotype was at least 75 kb (chromosome 21: chr21:330,003,56-chr21:330,754,04) (Table 3). The *SOD1*-p.H47R families and family 8 shared a common haplotype which was consistent with the North American *SOD1*-p.A5V haplotype. However, the shared haplotype in family 6, 7, 9 and 10 was the same as the *SOD1*-p.A5V haplotype found in the Swedish population and Iberian population (Table 4).

**Table 3.** Genotypes of genetic markers surrounding *SOD1* in patients and available family members.

RS number	Position (GRCh37)	MAF in East Asian	Family 2		Family 4						Family 6						Family 7			Family 8							Family 9		Family 10		
			Proband 2		Proband 4		III-1		III-2		Proband 6		II-6		III-1		Proband 7		I-2	Proband 8			III-1		IV-1		Proband 9		Proband 10		
rs999106	chr21:32968981	0.53	C	A	C	C	A	C	C	C	A	A	C	A	C	A	A	A	A	C	A	C	A	C		C	C	C	A	A	A
rs2070422	chr21:33000356	0.60	T	T	T	T	T	T	T	T	C	C	T	C	T	C	T	C	T	T	C	T	C	T		T	T	C	C	C	C
rs4816405	chr21:33033001	0.49	G	G	G	G	G	G	G	G	G	C	G	G	G	G	G	C	G	G	C	G	C	G		G	G	C	C	C	C
rs121912443 (p.H47R)	chr21:33036170	.	A	G	A	G	A	G	A	A	A	A	A	A	A	A	A	A	A	A	A	A	A	A		A	A	A	A	A	A
rs1568809169 (p.V48A)	chr21:33036173	.	T	T	T	T	T	T	T	T	T	C	T	T	T	T	T	C	T	T	T	C	T	C		T	C	T	C	T	C
rs2070424	chr21:33039320	0.46	G	G	G	G	G	G	G	G	G	A	G	G	G	G	G	A	G	G	A	G	A	G		G	G	A	A	A	A
rs2833475	chr21:33044820	0.49	G	G	G	G	G	G	G	G	G	A	G	G	G	G	G	A	G	G	A	G	A	G		G	G	A	A	A	A
rs16988427	chr21:33050785	0.46	C	C	C	C	C	C	C	C	C	T	C	C	C	C	C	T	C	C	T	C	T	C		C	C	T	T	T	T
rs2833481	chr21:33062351	0.49	C	C	C	C	C	C	C	C	T	T	T	C	T	C	C	T	C	C	T	C	T	C		C	C	T	T	T	T
rs2070423	chr21:33063354	0.45	T	T	T	T	T	T	T	T	C	C	C	T	C	T	T	C	T	T	C	T	C	T		T	T	C	C	C	C
rs2833483	chr21:33075404	0.49	C	C	C	C	C	C	C	C	C	T	C	C	C	C	C	T	C	C	T	C	T	C		C	C	T	T	T	T
rs7283466	chr21:33193131	0.79	G	G	A	G	G	G	A	G	G	G	A	G	G	A	G	G	G	A	G	G	G	G		G	G	G	A	G	A
rs2833556	chr21:33285300	0.74	G	G	A	G	A	G	A	A	G	G	A	G	G	G	G	G	G	A	A	A	A	A		G	A	A	G	A	G
rs8134939	chr21:33409932	0.69	C	T	T	C	C	C	C	C	C	T	C	C	C	T	T	C	T	C	T	C	C	C		C	C	T	T	C	T
rs2833640	chr21:33450081	0.34	A	A	G	A	G	A	A	G	A	A	A	G	A	A	A	A	A	G	G	A	G	A		A	A	A	G	A	A

MAF: minor allele frequency.

Marked with yellow and blue shading – haplotypes of *SOD1* p.H47R and p.V48A (indicated in red type-face).

**Table 4.** *SOD1*-p.H47R or p.V48A mutation founder haplotype in the Chinese population compared to other populations.

RS number	Reference to alternative allele	*SOD1-*p.A5V haplotype				*SOD1-*p.H47R or p.V48A haplotype						
		USA^a^	SWE^a^	CHN^a^	IBS^a^	Family 2	Family 4	Family 6	Family 7	Family 8	Family 9	Family 10
rs2070422	C > T	T	C	C	C	T	T	C	C	T	C	C
rs4816405	C > G	G	C	C/G	C	G	G	C	C	G	C	C
rs2070424	A > G	G	A	A	A	G	G	A	A	G	A	A
rs2833475	A > G	G	A	A	A	G	G	A	A	G	A	A
rs16988427	T > C	C	T	T/C	T	C	C	T	T	C	T	T
rs2833481	T > C	C	T	T	T	C	C	T	T	C	T	T
rs2070423	C > T	T	C	C/T	C	T	T	C	C	T	C	C
rs2833483	T > C	C	T	T/C	T	C	C	T	T	C	T	T
rs7283466	G > A	–	–	–	–	G	G	–	–	G	–	–

USA: the United States of America; SWE: Sweden; CHN: China; IBS: IBerian populations in Spain; (–) not available.

^a^ Data from Garcia et al. [49].

## Discussion

*SOD1* variants are notably prevalent among Asian population and more than 200 variants have been reported by now. However, *SOD1*-ALS patients presented with a remarkable diversity in clinical presentation. Our collective research, spanning past and present investigations, has identified 44 probands carrying 25 *SOD1* variants, of which p.H47R, p.V48A and p.C112Y variants were the most frequent. In addition, the clinical features of the patients carrying these three variants were described.

In our cohort, five probands were identified to carry *SOD1* p.H47R variant, who predominantly displayed LMN features and prolonged disease course with intact bulbar function. Five other probands with *SOD1* p.V48A variant exhibited limb onset, and 80% of them developed bulbar function impairment in the subsequent disease course. LMN impairment was common in most patients. Additionally, five another probands were detected to carry *SOD1* p.C112Y variant. All probands clinically presented with limb onset and prominent LMN signs. Bulbar symptoms were also frequently observed among them. Combined with the literature review results, there were 34, 11 and 18 pedigrees carrying p.H47R, p.V48A and p.C112Y variants, respectively. Remarkably, almost a quarter of the pedigrees were sporadic, which is potentially attributable to incomplete penetrance or lack of clear inadequate family medical history. Patients with p.H47R variant typically exhibited lower limb onset with predominantly LMN features and a prolonged duration, which could be easily misdiagnosed as Charcot-Marie-Tooth at the early stage. Most patients with p.V48A had limb onset. The disease duration in most patients was between 2 and 5 years. Patients with p.C112Y variant showed remarkable phenotypic variation, evidenced by a broad range of onset ages and variable disease progression patterns.

As the amino acid His47 acts as a copper ligand, the p.H47R disturbs copper binding and weakens affinity to zinc resulting in destabilized, toxic aggregation [41]. In a transgenic mouse model expressing p.H47R mutant SOD1, researchers observed hindlimbs muscle weakness and atrophy, along with SOD1 and ubiquitin-positive aggregates in the anterior horns [42]. Moreover, these mice displayed structural alterations in the cristae of spinal cord mitochondria [43]. The Val48 residue sits at a β-turn and between two copper ion-binding sites, His47 and His49 [44], highlighting its potential influence on SOD1 structure and function. A substitution from valine to alanine results in the introduction of a smaller branch chain which may interrupt the copper ion binding and lead to the accumulation of a cytotoxic SOD1 aggregated species [45]. The C112 residue is cysteine and the p.C112Y variant has a heightened the propensity for aggregation [39]. Liu et al. found the mitochondrial impairments in fibroblast and iPSC derived from the patient carrying p.C112Y variant [33,36].

As functional studies had been performed in SOD1^H47R^ mice and SOD1^C112Y^ fibroblast and iPSC, our focus shifted to elucidating cellular dysfunction caused by the p.V48A variant. We found that this variant induces the aggregation of SOD1 protein in *SOD1^V48A^* fibroblast compared to control fibroblast. Additionally, the accumulation of mutated SOD1 proteins is consistently implicated in multifaceted mitochondrial dysfunction in ALS, such as axonal transport inhibition, energy deficiency and increased cellular ROS production [46,47]. Considering the fundamental role of mitochondria in cellular metabolism and energy generation, their dysfunction in ALS may cause motor neuron death through calcium-mediated excitotoxicity, increasing ROS and activation of intrinsic apoptotic pathway [48,49]. In this study, we measure the intracellular ROS level and MMP to assess mitochondrial impairments. Compared to the control fibroblast, the elevated ROS level and decreased MMP were observed in *SOD1^V48A^* fibroblasts, which was consistent with this line of evidence.

As shown in our previous study, the p.H47R, p.V48A and p.C112Y variants were common variants [16]. Of note, the p.H47R and p.C112Y variants are mainly distributed in Asia; the p.V48A variant has been exclusively documented in individuals of Chinese descent. Considering the proximity of these two loci, a common founder has been hypothesized for these two variants. In this study, haplotype analysis of seven families showed two distinct allele configurations. As *SOD1* p.A5V variant resides 4 kb upstream from p.H47R and p.V48A variants, recombination at this genomic region is deemed unlikely. We assumed the *SOD1*-p.A5V haplotype was comparable with the *SOD1*-p.H47R and *SOD1*-p.V48A haplotypes. Our analysis revealed a fascinating pattern: the haplotypes in four families matched the typical European *SOD1*-p.A5V haplotype, while another family’s haplotype aligned with the American haplotype profile. This finding resonates with the work by Garcia et al. which revealed that the American haplotype is most prevalent in East Asian populations, succeeded by the European haplotype. These observations offer valuable insights into the geographic distribution and potential migratory histories of these genetic lineages [49]. However, current data showed *SOD1*-p.V48A is largely associated with the European haplotype, while the *SOD1*-p.H47R aligns with the American haplotype. This diverges from our hypothesis that these prevalent Chinese variants share a unitary ancestral origin. Therefore, a new hypothesis posits dual (North American and European) ancestries for the p.V48A variant, which needed more SOD1 p.V48A samples to validate. Despite the American association of the p.H47R haplotype, its presence in a scattering of Japanese, European and American patients hints at a more intricate, globally dispersed heritage (Supplementary Table S3). To delve deeper into the origins of the SOD1 p.H47R variant, a meticulous haplotype analysis among affected ALS patients is imperative. This endeavour would benefit immensely from an internationally coordinated effort, where a consortium collaborates to recruit and meticulously examine ALS families globally.

There are some limitations in this study. First, the sample size of *SOD1* p.H47R or p.V48A patients was small and the blood sample of family members was not collected completely, which hindered comprehensive haplotype interpretation. Expanding the pool of p.H47R and p.V48A patients is essential to robustly discern any shared ancestral roots for these variants in Asian ALS populations. Another limitation lies in the inability to adequately conduct haplotype assessments for *SOD1* p.C112Y patients due to a scarcity of familial samples. This investigation further illuminated the p.V48A variant’s role in mitochondrial dysfunction and cytotoxic aggregation formation. Since *SOD1* variants also implicate pathways like endoplasmic reticulum stress and protein homeostasis, additional research is imperative to unravel the complex mechanisms at play in *SOD1*-ALS using patient-derived fibroblasts.

In conclusion, we reported the Chinese ALS patients with *SOD1* p.H47R, p.V48A or p.C112Y variant and summarized the genotype–phenotypes of p.H47R, p.V48A and p.C112Y patients. We further demonstrated mitochondrial disturbances and mutant SOD1 aggregation in SOD1V48A fibroblasts. Our research enhances comprehension of the genotype–phenotype relationship in ALS cases linked to SOD1 variants and disputes the notion of a common founder effect for p.H47R and p.V48A variants.

## Supplementary Material

Supplemental Material

## Data Availability

The data are available upon a reasonable request from the corresponding author.
