# Supplementary material for: Clinical characterization and founder effect analysis in Chinese amyotrophic lateral sclerosis patients with SOD1 common variants
Source: Ann Med. 2024 Oct 1;56(1):2407522. doi: 10.1080/07853890.2024.2407522 (PMC11445911; doi:10.1080/07853890.2024.2407522)
Supplement: Supplemental Material [file IANN_A_2407522_SM1438.zip › Supplemental Material/Supplementary Table S1 (2).docx]

**Supplementary Table S1.** Primers for the 15 SNPs.

| **SNP** | **Forward Primer** | **Reward Primer** |
| --- | --- | --- |
| rs999106 | CCCACTGATTCTACATTACGG | TCCTATCCACATGACTGCA |
| rs2070422 | ACAGAACCTGTGTGGCCATT | TGTGCAGCAGAGCTGGTATG |
| rs4816405 | CAAGGGCAGAGAAGGTAGCC | CACCCACTGTCCTCAAACGA |
| rs121912443 | GCCTGGGATTTGGACACAGA | GGGGTTTTAACGTTTAGGGGC |
| rs1568809169 | GCCTGGGATTTGGACACAGA | GGGGTTTTAACGTTTAGGGGC |
| rs2070424 | GCTCCCAAATGCTGGAATGC | GGATCTTTAGAAACCGCGACT |
| rs2833475 | CCGAAGATGGCACTCCAGTT | TGTGCACCTGCAGTATGCTT |
| rs16988427 | ACAGCATCATATTCTTAGCTGCC | AGGGAAGGCATCTGAACCAG |
| rs2833481 | TGTTCTTTCTGAGGGCGGTG | CCTTGGAAAGTTTTAGATGTCCAGA |
| rs2070423 | TGCAAGGAACAGGACATTCG | ACAAGGCACCCCTAGACTTG |
| rs2833483 | AGCAACTGGCCTGTAGTCAA | TGCAGTAGGAAAAGACAGCGA |
| rs7283466 | AAGGCTGACAAACACCGCC | TCCTTGGGTGCCTCTTGTC |
| rs2833556 | CGGTGTCCACAGATAGCAC | ATACCCTCCACTGTACTTTCC |
| rs8134939 | AGAGCGCACAATAGCAGCA | AGCCCCAGATGCAGTTTGT |
| rs2833640 | GCTCTACCTAATTGAGTGG | GACACTCTGTTTAAGGATG |
