# Supplementary material for: Clinical characterization and founder effect analysis in Chinese amyotrophic lateral sclerosis patients with SOD1 common variants
Source: Ann Med. 2024 Oct 1;56(1):2407522. doi: 10.1080/07853890.2024.2407522 (PMC11445911; doi:10.1080/07853890.2024.2407522)
Supplement: Supplemental Material [file IANN_A_2407522_SM1438.zip › Supplemental Material/Supplementary Table S2.docx]

**Supplementary Table S2.** Clinical features of the patients carrying *SOD1* variants in our cohort.

| Exon | Nucleotide change | Protein change | Case count (n) | Male/Female | FALS/SALS | Spinal onset | AAO, year, mean ± SD | Diagnostic delay, month, median (range) | Survival time, month, mean ± SD |
| --- | --- | --- | --- | --- | --- | --- | --- | --- | --- |
| 1 | c.13G>T | p.A5S | 1 | 1/0 | 1/0 | 1 | 29 | 3 | 12 |
| 1 | c.14C>T | p.A5V | 1 | 0/1 | 1/0 | 1 | 60 | 8 | 15 |
| 1 | c.20G>T | p.C7F | 1 | 0/1 | 0/1 | 1 | 45 | 7 | 10 |
| 1 | c.32G>T | p.G11V | 1 | 0/1 | 1/0 | 1 | 25 | 9 | 15 |
| 1 | c.49G>T | p.G17C | 1 | 1/0 | 1/0 | 1 | 49 | 8 | 38 |
| 1 | c.49_50delinsCA | p.G17H | 1 | 1/0 | 1/0 | 1 | 23 | 4 | 36 |
| 1 | c.62T>G | p.F21C | 1 | 1/0 | 1/0 | NA | NA | NA | NA |
| 1 | c.112G>C | p.G38R | 1 | 1/0 | 1/0 | 1 | 26 | 36 | 156^a^ |
| 2 | c.140A>G | p.H47R | 5 | 1/4 | 5/0 | 5 | 53.4 ± 3.2 | 18 (13-84) | 113.4 ± 60.5^aaaaa^ |
| 2 | c.143T>C | p.V48A | 5 | 2/3 | 5/0 | 5 | 50.8 ± 8.4 | 13 (12-20) | 35.2 ± 14.8^aaa^ |
| 3 | c.223C>T | p.P75S | 1 | 1/0 | 0/1 | 1 | 58 | 23 | 65 |
| 4 | c.251A>G | p.D84G | 1 | 1/0 | 1/0 | 1 | 32 | 1 | 70 |
| 4 | c.255G>C | p.L85F | 1 | 0/1 | 1/0 | 1 | 52 | 108 | 110^a^ |
| 4 | c.260A>G | p.N87S | 3 | 2/1 | 1/2 | 3 | 54.0 ± 14.2 | 11 (4-11) | 14.0 ± 4.6^aa^ |
| 4 | c.317C>T | p.S106L | 1 | 1/0 | 0/1 | 1 | 49 | 5 | 27^a^ |
| 4 | c.319C>G | p.L107V | 1 | 1/0 | 1/0 | 1 | 41 | 2 | 10 |
| 4 | c.335G>A | p.C112Y | 5 | 5/0 | 4/1 | 5 | 44.8 ± 9.1 | 9 (6-15) | 37.8 ± 19.5^aa^ |
| 4 | c.341T>C | p.I114T | 1 | 1/0 | 0/1 | 1 | 60 | 10 | 21 |
| 5 | c.363T>G | p.H121Q | 3 | 1/2 | 2/1 | 3 | 48.3 ± 10.1 | 8 (7-11) | 38.3 ± 19.5^a^ |
| 5 | c.400G>T | p.E134* | 1 | 1/0 | 1/0 | 1 | 48 | 6 | 30 |
| 5 | c.412A>G | p.T138A | 1 | 1/0 | 0/1 | 1 | 45 | 143 | 157^a^ |
| 5 | c.434T>C | p.L145S | 2 | 0/2 | 2/0 | 2 | 49.0 ± 1.0 | 17 (6-34) | 59.0 ± 8.0^aa^ |
| 5 | c.439T>C | p.C147R | 1 | 0/1 | 1/0 | 0 | 39 | 3 | 9 |
| 5 | c.443G>A | p.G148D | 3 | 0/3 | 3/0 | 3 | 36.3 ± 2.1 | 6 (4-12) | 13.7 ± 1.5 |
| 5 | c.449T>C | p.I150T | 1 | 0/1 | 1/0 | 1 | 37 | 7 | 12 |

FALS: familial amyotrophic lateral sclerosis; SALS: Sporadic amyotrophic lateral sclerosis; AAO: age at onset; NA: not available.

^a^There was one surviving case. ^aa^There were two surviving cases. ^aaa^There were three surviving cases. ^aaaaa^There were five surviving cases.
