# Supplementary material for: Clinical characterization and founder effect analysis in Chinese amyotrophic lateral sclerosis patients with SOD1 common variants
Source: Ann Med. 2024 Oct 1;56(1):2407522. doi: 10.1080/07853890.2024.2407522 (PMC11445911; doi:10.1080/07853890.2024.2407522)
Supplement: Supplemental Material [file IANN_A_2407522_SM1438.zip › Supplemental Material/Supplementary Table S3.docx]

**Supplementary Table S3.** Clinical features of patients carried *SOD1* p.H47R.

| Pedigree | Familial distribution | Country | Family history | Gender | AAO, year, mean ± SD (n) | Onset site (n) | Diagnostic delay, month, median (range) | Phenotype | Bulbar symptom | Respiratory failure | Cognitive impairment | Disease duration, year, mean ± SD (n) | Reference |
| --- | --- | --- | --- | --- | --- | --- | --- | --- | --- | --- | --- | --- | --- |
| P1 | Proband | China | Y | F | 58 | UL | 84 | LMN dominance | - | NA | - | >15 | Present study |
| P2 | Proband | China | Y | M | 53 | LL | 18 | LMN dominance | - | - | - | >10.4 | Present study |
| P3 | Proband | China | NA | F | 55 | LL | 18 | LMN dominance | NA | NA | NA | >12 | Present study |
| P4 | Proband | China | Y | F | 50 | UL | 45 | Classical ALS | - | - | - | >8.3 | Present study |
| P5 | Proband | China | Y | F | 51 | LL | 13 | LMN dominance | - | - | - | >1.6 | Present study |
| P6 | 10 patients in 2 generations | China | Y | 2M/4F | 49.4±9.4(10) | LL (10) | NA | LMN dominance | - | 3 | - | 16.5±13.9 (10) | Li et al.[18] |
| P7 | 4 patients in 2 generations | China | Y | 2M/2F | 51.0±11.1(4) | LL(4) | NA | LMN dominance | - | - | NA | 9.5±1.9 (4) | Zou et al.[19] |
| P8 | Proband | China | N | F | 41 | LL | NA | Classical ALS | - | - | - | >9 | Zou et al.[19] |
| P9 | Proband | China | N | M | 55.1 | NA | NA | NA | NA | NA | NA | >23.8 | Chen et al.[20] |
| P10 | Proband | China | Y | F | 43.3 | NA | NA | NA | NA | NA | NA | >50.1 | Chen et al.[20] |
| P11 | Proband | China | Y | M | 34.1 | NA | NA | NA | NA | NA | NA | >105.5 | Chen et al.[20] |
| P12-19 | 8 Probands | China | 6Y/2N | 3M/5F | 51.5±3.5(8) | NA | 55.5(11-144) | NA | NA | NA | NA | 96.0±50.5 (8) | Tang et al.[21] |
| P20 | 13 patients in 4 generations | Japan | Y | 4M/9F | 49.6±10.9 (10) | LL (6) | NA | LMN dominance | 1 | - | - | 17.3±10.7 (4) | Aoki et al.[22] |
| P21 | 15 patients in 4 generations | Japan | Y | 6M/9F | 4S.0±9.5(14) | LL (1) | NA | Classical ALS | - | NA | NA | 16.8±6.8 (9) | Aoki et al.[22] |
| P22 | 11 patients in 4 generations | Japan | Y | 7M/4F | 9.7±10.5(9) | LL (9) | NA | LMN dominance | 1 | 1 | NA | 8.1±13.2 (9) | Ohi et al.[23] |
| P23 | 17 patients in 4 generations | Japan | Y | 7M/10F | 4.3±8.7(17) | LL (17) | NA | LMN dominance | 1 | 7 | - | 12.1±7.6 (17) | Arisato et al.[24] |
| P24 | 15 patients in 4 generations | Japan | Y | 5M/10F | 42.9±4.7(7) | LL (7) | NA | LMN dominance | - | - | NA | 17.2±8.1 (7) | Ohi et al.[25] |
| P25 | 3 patients in 2 generations | Japan | Y | 1M/2F | 49.3±5.8(3) | LL (3) | NA | LMN dominance | - | - | 1 | 25.0±13.0 (2) | Yamashita et al.[26] |
| P26 | 4 patients in 3 generations | French | Y | 3M/1F | NA | LL (1) | NA | LMN dominance | - | 2 | NA | 11.5±10.6 (4) | Camu et al.[27] |
| P27 | 3 patients in 3 generations | Norway | Y | 1M/2F | 43.3±15.3(3) | LL (3) | NA | LMN dominance | - | - | - | 19.0±26.9 (3) | Holmøy et al.[28] |
| P28 | 22 patients in 6 generations | Norway | Y | 10M/ 12F | 42.5±11.0 (22) | LL (20) | NA | LMN dominance | - | - | - | 10.6±13.1 (21) | Østern et al.[29] |
| P29 | Proband | Pakistan | N | M | 55 | LL | 20 | Classical ALS | - | - | - | >20 | Holmøy et al.[30] |
| P30-34 | 5 probands | USA | NA | NA | 43.2±11.7(5) | NA | NA | NA | NA | NA | NA | 17.4±6.4 (5) | Juneja et al.[10] |

AAO: age at onset; M: male; F: female; SD: standard deviation; Y: yes; N: no; LL: lower limb; UL: upper limb; LMN: lower motor neuron; ALS: amyotrophic lateral sclerosis; NA: not available..
